# Sea Anemone *Heteractis crispa* Actinoporin Demonstrates In Vitro Anticancer Activities and Prevents HT-29 Colorectal Cancer Cell Migration

**DOI:** 10.3390/molecules25245979

**Published:** 2020-12-17

**Authors:** Aleksandra Kvetkina, Olesya Malyarenko, Aleksandra Pavlenko, Sergey Dyshlovoy, Gunhild von Amsberg, Svetlana Ermakova, Elena Leychenko

**Affiliations:** 1 G.B. Elyakov Pacific Institute of Bioorganic Chemistry, Far Eastern Branch, Russian Academy of Sciences, 159, Pr. 100 let Vladivostoku, Vladivostok 690022, Russia; malyarenko.os@gmail.com (O.M.); apavlenko141@gmail.com (A.P.); swetlana_e@mail.ru (S.E.); leychenko@gmail.com (E.L.); 2 Department of Oncology, Hematology and Bone Marrow Transplantation with Section Pneumology, Hubertus Wald-Tumorzentrum, University Medical Center Hamburg-Eppendorf, 20251 Hamburg, Germany; dyshlovoy@gmail.com (S.D.); g.von-amsberg@uke.de (G.v.A.); 3 Martini-Klinik, Prostate Cancer Center, University Hospital Hamburg-Eppendorf, 20251 Hamburg, Germany; 4 School of Natural Sciences, Far Eastern Federal University, Vladivostok 690922, Russia

**Keywords:** actinoporin, sea anemone, *Heteractis crispa*, anticancer activity, anti-migratory activity

## Abstract

Actinoporins are the most abundant group of sea anemone cytolytic toxins. Their membranolytic activity is of high interest for the development of novel anticancer drugs. However, to date the activity of actinoporins in malignant cells has been poorly studied. Here, we report on recombinant analog of Hct-S3 (rHct-S3), belonging to the combinatory library of *Heteractis crispa* actinoporins. rHct-S3 exhibited cytotoxic activity against breast MDA-MB-231 (IC_50_ = 7.3 µM), colorectal HT-29 (IC_50_ = 6.8 µM), and melanoma SK-MEL-28 (IC_50_ = 8.3 µM) cancer cells. The actinoporin effectively prevented epidermal growth factor -induced neoplastic transformation of JB6 Cl41 cells by 34% ± 0.2 and decreased colony formation of HT-29 cells by 47% ± 0.9, MDA-MB-231 cells by 37% ± 1.2, and SK-MEL-28 cells by 34% ± 3.6. Moreover, rHct-S3 decreased proliferation and suppressed migration of colorectal carcinoma cells by 31% ± 5.0 and 99% ± 6.4, respectively. The potent anti-migratory activity was proposed to mediate by decreased matrix metalloproteinases-2 and -9 expression. In addition, rHct-S3 induced programmed cell death by cleavage of caspase-3 and poly (ADP-ribose) polymerase, as well as regulation of Bax and Bcl-2. Our results indicate rHct-S3 to be a promising anticancer drug with a high anti-migratory potential.

## 1. Introduction

Cancer is a major public burden with tens of millions people being diagnosed around the world every year. Eventually, more than half of the patients succumb to their disease despite new developments [1]. In 2018, 9.6 million people died from cancer according to the World Health Organization [2], with increasing numbers in developing countries. Lung, breast, colorectal, and prostate cancer belong to the most frequently diagnosed malignancies worldwide [2]. These diseases claim the lives of more than a million people annually.

Although scientific and technological progress allowed the development of new approaches such as gene-therapy [3], stem cell transplantation [4], immunotherapy [5], and therapy by nanoparticles [6] for cancer treatment, the traditional cancer therapy including a combination of surgery, radio- and chemotherapy is still the most commonly used [7,8,9]. Standard cancer therapy is accompanied by various drawbacks, such as a lack of tumor-specific drug delivery systems, regular application of toxic anticancer drugs leading to adverse side effects, in particular normal cell death, drug resistance, as well as cancer recurrence after surgical removal of solid tumors [5,10]. Therefore, the search for more effective anticancer compounds is ongoing.

In this context, actinoporins, cytolytic toxins derived from sea anemones (marine venomous cnidarians), were identified as promising candidates for cancer therapy. This unique group of small basic α-pore-forming proteins includes a compact β-fold lacking disulfide bounds formed by 12 β-sheets and two α-helices—one of which, functional and more extended, is located at the N-terminus, and the second one, short, is at the C-terminus [11]. Cytotoxicity of actinoporins relies on the formation of pores within sphingomyelin-containing membranes, which disrupts ion gradients that lead to osmotic swelling and ultimately to cell death [12,13,14]. Cytolytic activity of actinoporins was observed in different cells including platelets, fibroblasts, parasite cells, lactotrophs and some cancer cells [15,16]. Due to pore-forming ability and selective binding to sphingomyelin on the cell membrane surface, high stability to temperature and proteolytic cleavage, they are currently considered as antibacterial and anticancer agents as well as components of immunotoxins [17,18]. These immunotoxins include StnI from *Stichodactyla helianthus* [17], Gigantoxin-4 from *Stichodactyla gigantea* [19], EqII from *Actinia equina* [20] and its mutant EqTx-II(I18C) [21], as well as FraC from *Actinia fragacea* [22].

In our previous studies, we reported the structure and first functional analyses of actinoporins isolated from *Heteractis crispa* (=*Radianthus macrodactylus*) [23,24,25,26,27,28]. We were able to demonstrate cytotoxic activity of actinoporin RTX-A in monocytic leukemia (THP-1) cells, cervix carcinoma (HeLa), breast (MDA-MB-231) and colon (SNU-C4) cancer cells. In addition, epidermal growth factor (EGF)-induced tumor transformation of mouse epidermal JB6 P^+^ Cl41 cells was observed. Activity was mediated by induction of p53-independent apoptosis as well as inhibition of the oncogenic nuclear factors AP-1 and NF-κB [16]. Moreover, we found the combinatorial library of *H. crispa* actinoporins encoded by the multigene family including at least 47 representatives [26,27,28]. Here, we report the in vitro anticancer activity of the recombinant analog of Hct-S3, the most abundant isoform of *H. crispa* actinoporins.

## 2. Results

### 2.1. Obtaining of Recombinant Analog of Hct-S3 Actinoporin

**Figure 1:**
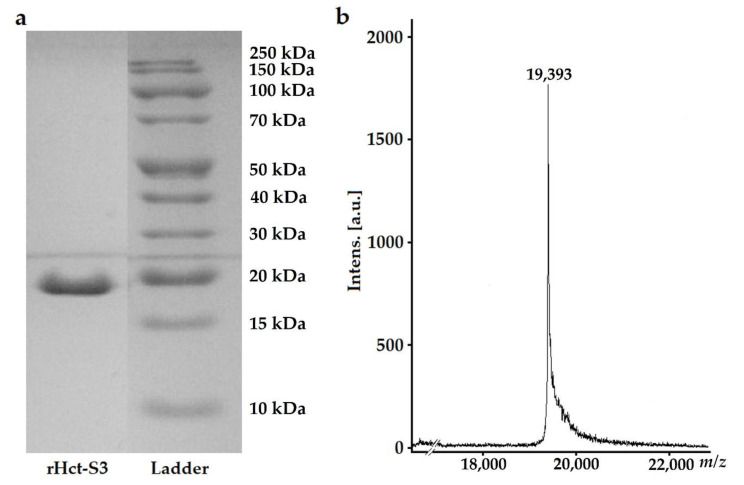
Electrophoregram (**a**) and mass spectrum (**b**) of purified rHct-S3.

Hct-S3 (177 amino acid residues) is one of the most represented isoforms belonging to the multigene family of *H. crispa* actinoporins. A recombinant analog of Hct-S3 (rHct-S3) was expressed in *Escherichia coli* strain Rosetta (DE3) as fusion protein containing glutation-S-transpherase, polyhistidine tag, enteropeptidase cleavage site, and actinoporin. In order to avoid the denaturation of the target polypeptide and increase its yield, we applied a high-pressure homogenization approach using a French-press homogenizer for the cell destruction instead of ultrasonication. The fusion protein with a molecular mass of ~50 kDa was isolated using a metal-affinity chromatography and cleaved by enteropeptidase. Next, the targeted actinoporin was purified on a soybean trypsin inhibitor -affinity column and further desalted (Figure 1a). The final yield of rHct-S3 after high-pressure homogenization was 1 mg/L of cell culture in contrast to 0.2 mg/L yield after ultrasonication. The molecular mass of the polypeptide was determined by MALDI-TOF/TOF mass-spectrometry as 19,393 Da (Figure 1b), which is consistent with the predicted molecular mass (19,390 Da). The N-terminal amino acid sequence (15 aa) determined by the automated Edman degradation matched well with the amino acid sequence deduced from cDNA earlier.

### 2.2. The Effect of rHct-S3 on Cell Viability

**Figure 2:**
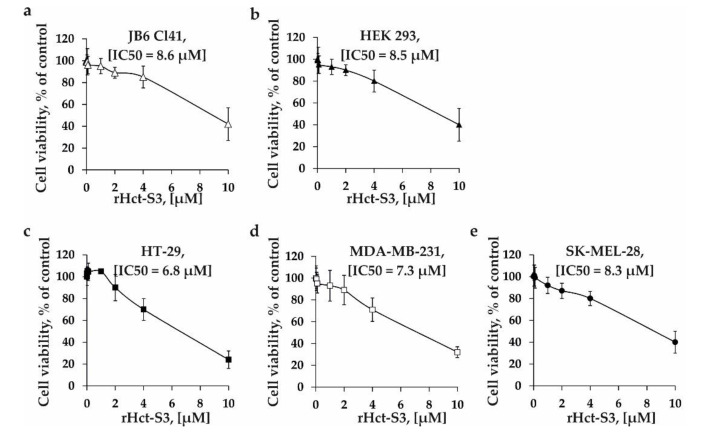
The effect of rHct-S3 on cell viability of (**a**) normal mouse epidermal cells JB6 Cl41, human (**b**) embryonic kidney HEK 293, (**c**) colorectal carcinoma HT-29, (**d**) breast cancer MDA-MB-231, and (**e**) melanoma SK-MEL-28 cell lines. The cytotoxic activity was determined by MTS assay after 24 h of treatment. The results are expressed as the percentage of inhibition that produced a reduction in absorbance by rHct-S3 treatment compared the non-treated cells. Results are expressed as the mean ± standard deviation (SD).

In order to determine the cytotoxic effect of rHct-S3, the panel of human cancer cell lines HT-29 (colorectal carcinoma), MDA-MB-231 (triple negative breast cancer), SK-MEL-28 (malignant melanoma), as well as normal mouse epidermal JB6 Cl41 cells and human embryonic kidney HEK 293 cells were treated by rHct-S3 at a concentration range 0.01 µM–10 µM for 24 h and cell viability was estimated by MTS assay. rHct-S3 had comparable effects on viability of cell lines with an IC_50_ of 8.6 µM for JB6 Cl41 cells (Figure 2a), 8.5 µM for HEK 293 (Figure 2b), 6.8 µM for HT-29 cells (Figure 2c), 7.3 µM for MDA-MB-231 cells (Figure 2d), and 8.3 µM for SK-MEL-28 cells (Figure 2e).

### 2.3. The Effect of rHct-S3 on EGF-Induced Neoplastic Transformation of Normal Cells and Colony Formation of Cancer Cells

**Figure 3:**
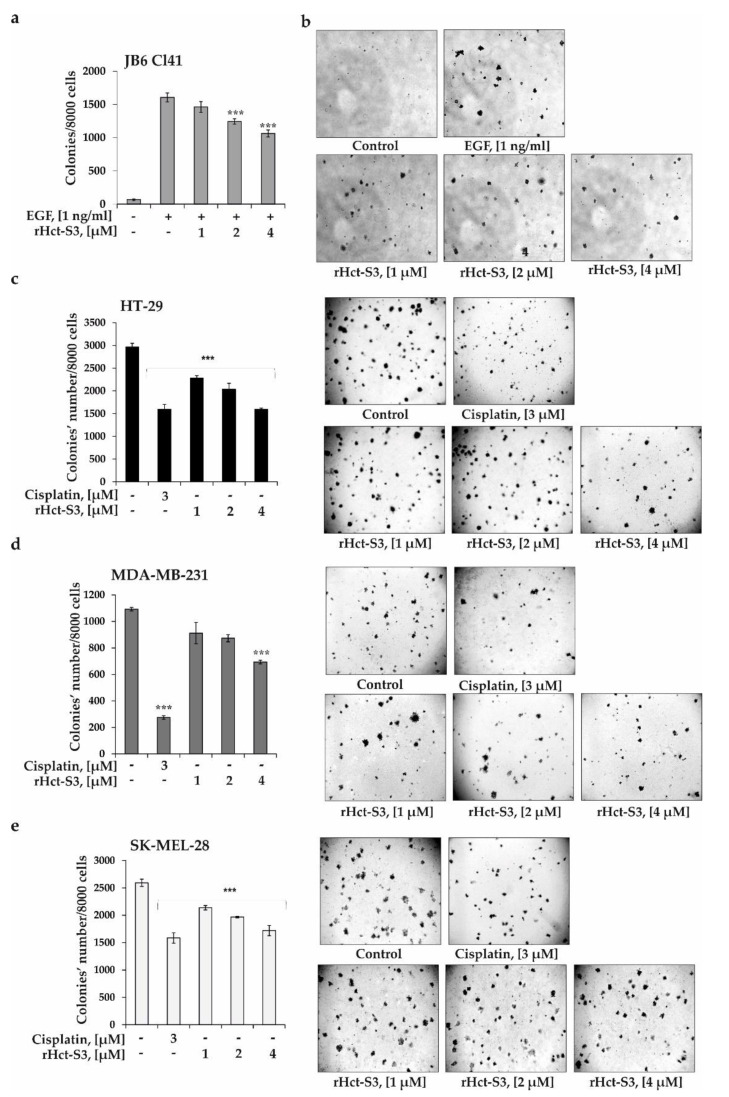
Effect of rHct-S3 on EGF-induced neoplastic cells transformation of JB6 Cl41 cells and colony formation of human colorectal carcinoma HT-29, breast cancer MDA-MB-231, and melanoma SK-MEL-28 cell lines. (**a**,**b**) JB6 Cl41 cells (2.4 × 10^4^ /mL) treated with/without EGF (1 ng/mL) or investigated compound (1, 2, and 4 µM) in 1 mL of 0.3% Basal medium Eagle (BME) agar containing 10% FBS and overlaid with 3.5 mL of 0.5% BME’s agar containing 10% FBS. The culture was maintained at 37 °C in a 5% CO_2_ atmosphere for 2 weeks. (**c**) HT-29, (**d**) MDA-MB-231, (**e**) SK-MEL-28 cells (2.4 × 10^4^ /mL) treated with/without investigated compound (1, 2, and 4 µM) or cisplatin at 3 µM (positive control) and subjected into a soft agar. The culture was maintained at 37 °C in a 5% CO_2_ atmosphere for 2 weeks. The colonies were counted under a microscope with the aid of the ImageJ software program (*n* = 6 for control and each compound, *n*—quantity of photos). The magnification of representative photos of colonies is ×10. The asterisks (*** *p* < 0.001) indicate a significant decrease in colony formation in cells treated with compound compared with the non-treated cells (control).

The effects of rHct-S3 on neoplastic transformation of JB6 Cl41 cells induced by EGF, colony formation and growth of cancer cells were studied by soft agar assay, which is considered to be the most accurate type of in vitro test for detecting malignant transformation of cells [29]. The actinoporin was found to inhibit the EGF-induced neoplastic transformation of JB6 Cl41 cells by 10% ± 5.0, 23% ± 2.5, and 34% ± 0.2 at subtoxic concentrations of 1, 2, and 4 µM, respectively (Figure 3a,b). Moreover, rHct-S3 decreased the number of colonies of HT-29 cells by 25% ± 1.8, 33% ± 0.1, and 47% ± 0.9, at concentrations of 1, 2, and 4 µM, respectively, compared to non-treated cells (control) (Figure 3c). At the same doses, rHct-S3 inhibited the colony formation of MDA-MB-231 cells by 17% ± 2.4, 20% ± 2.5, 37% ± 1.2 and SK-MEL-28 cells by 18% ± 1.5, 24% ± 0.5, 34% ± 3.6, respectively (Figure 3d,e). Because activity of rHct-S3 was most pronounced in colorectal carcinoma HT-29 cells, further experiments were carried out with this cell line. It should be noted that chemotherapeutic drug, cisplatin, used as a positive control in this study, inhibited colony formation of HT-29, MDA-MB-231, and SK-MEL-28 cells by 46%, 75%, and 39% at a non-cytotoxic dose of 3 µM, respectively (Figure 3). These results indicate that the actinoporin has a promissing anticancer potential.

### 2.4. The Effect of rHct-S3 on Migration of Colorectal Carcinoma HT-29 Cells

**Figure 4:**
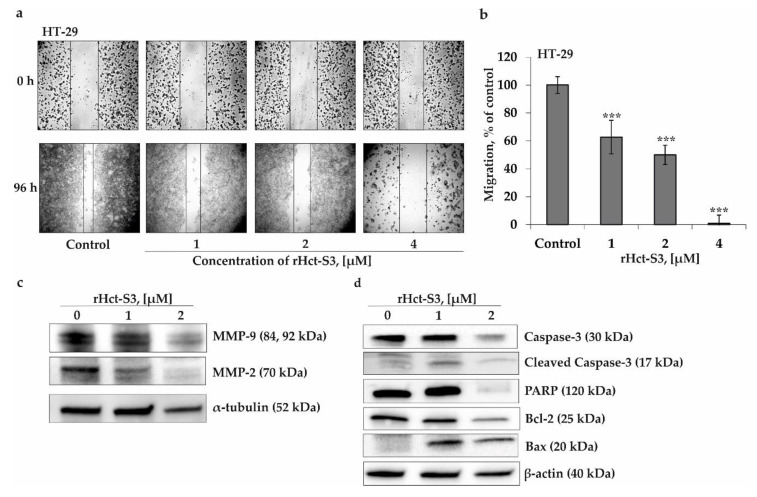
Effects of rHct-S3 on migration of HT-29 cells, MMPs and apoptotic proteins. (**a**,**b**) The HT-29 cells migration distance was measured the width of the wound and expressed as a percentage of each control for the mean of wound closure area. All experiments were repeated at least three times in each group (n = 18 for control and each compound, n—quantity of photos). The magnification of representative photos is ×10. The asterisks (*** *p* < 0.001) indicate a significant decrease in migration of cells treated with rHct-S3 compared with the non-treated cells (control) (**c**,**d**) rHct-S3 inhibited MMP-9, MMP-2 expression and regulated caspase-3, cleaved caspase-3, PARP, Bcl-2 and Bax in HT-29 cells, as determined by Western Blotting with specific antibodies.

We investigated the effect of rHct-S3 on the migration of colorectal carcinoma HT-29 cells with high metastatic potential, using a scratch assay. It was demostrated that rHct-S3 supressed migration of HT-29 cells by 33% ± 10.2, 50% ± 7.5, and 99% ± 6.4, respectively, at concentrations of 1, 2, and 4 µM, compared to the control group (Figure 4a,b). In order to reveal the impact of inhibition of proliferation by rHct-S3 on migration, the antiproliferative activity of rHct-S3 against HT-29 cells was checked in 24, 48, 72, and 96 h of treatment (Supplementary Figure S1). It was found that rHct-S3 at concentrations 1, 2, and 4 µM slightly (not more than 10%) decreased the proliferation rate of HT-29 cells after 24 h and 48 h of treatment, while it inhibited cells proliferation by 11% ± 3.0, 26% ± 1.2, and 31% ± 5.0, respectively, after 96 h of treatment. These results indicate that rHct-S3 possess a moderate antiproliferative activity.

To elucidate the potential mechanism of this anti-migratory activity, we evaluated the effect of rHct-S3 on the expression level of the matrix metalloproteinases (MMP)-2 and MMP-9, playing a pivotal role in cancer cell invasion and metastasis. Indeed, actinoporin effectively inhibited the expression of MMP-2 and MMP-9 (Figure 4c) at a concentration of 2 μM. In addition, we estimated whether rHct-S3 affect the activation of caspase-3, a known executor of apoptosis. The upregulation of cleaved caspase-3 was detected in HT-29 cells treated with rHct-S3. Additionally and in line with this, we have detected a degradation of poly (ADP-ribose) polymerase (PARP) as well as Bcl-2 down-regulation and Bax up-regulation (Figure 4d). Thus, rHct-S3 decreases the migratory activity of colorectal carcinoma HT-29 cells by the inhibition of MMP-2 and MMP-9 and induces the apoptosis via the activation of caspase-3.

## 3. Discussion

**Figure 5:**
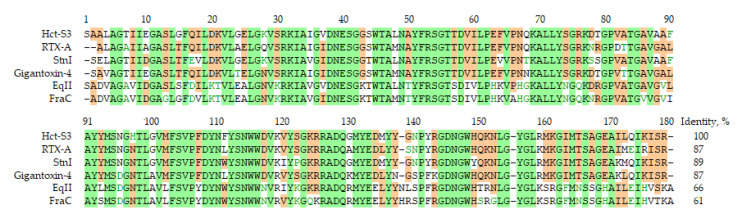
Multiple sequence alignment of actinoporins. EqII (P61914) from *Actinia equina*, FraC (B9W5G6) from *Actinia fragacea*, Gigantoxin-4 (H9CNF5) from *Stichodactyla gigantea*, StnI (P81662) from *Stichodactyla helianthus*, and RTX-A (P58691) from *Heteractis crispa*. The identical and conservative amino acid residues are shown on green and brown background.

Actinoporins are the major components of sea anemone venom, which disrupt cell membranes by pore formation [30]. *H. crispa* venom contains numerous actinoporin isoforms, encoded by the multigene family [28]. Hct-S3 is one of the isoform of *H. crispa* actinoporins belonging to Hct-S group with Ser at N-terminus (Figure 5). Earlier, the recombinant analog of Hct-S3 was obtained [26,31] and its hemolytic activity was comparable with well-characterized actinoporins such as RTX-A from *H. crispa* [32], EqII from *A. equina* [33] and StnII from *S. helianthus* [34]. Comparative analysis of amino acid sequences of known actinoporins and Hct-S3 revealed that Hct-S3 shared 87–89% identity with Gigantoxin-4 from *S. gigantea*, and RTX-A and StnI from *S. helianthus*, which possess anticancer activity (Figure 5). However, their anticancer mechanism has not been studied in detail. We attempted to elucidate the mechanism of action of actinoporins, in particular, Hct-S3, in different human cancer cells.

The recombinant analog of Hct-S3 was obtained using a previously developed scheme [26] with a changing of cell disruption approach. The lack of carbohydrates and disulfide bridges simplifies the production of recombinant actinoporins by heterologous expression in *E. coli*. However, there are some difficulties with the isolation of soluble actinoporins due to the protein aggregation as inclusion bodies during cells’ ultrasonication. It is known that ultrasonic homogenization is a high-energy process of cell disruption. This fact may lead to the samples heating and result in the denaturation of proteins. Therefore, to minimize the protein denaturation we used a high-pressure homogenization of *E. coli* cells that allow us to increase the yield of soluble form of rHct-S3 by five times.

Cytotoxic effects of rHct-S3 were studied in normal mouse epidermal, human embryonic kidney cells and human colon carcinoma, breast cancer, and melanoma cells. rHct-S3 exhibited cytotoxic activity against all tested cell lines with comparable IC_50_ values (Figure 2), which were 100–1000-fold higher than those found for other actinoporins [17,18,35]. Previously, StnI and hemolytic fraction of *S. helianthus* were shown to possess cytotoxic activities against colorectal cancer or breast cancer cells, respectively, while RTX-A demonstrated potent cytotoxic activity against both tested cancer cell lines [16].

Carcinogenesis is known to be a multistage process, which includes the initiation (transformation of normal cells into cancer cells), development (formation of colonies of cancer cells) and progression (growth of colonies of cancer cells) of cancer. Cancer prevention is gaining increasing attention because it may be a promising alternative to cancer treatment sparing complications caused by advanced diseases. The involvement of multiple factors and developmental stages and our increased understanding of cancer at the epigenetic, genetic, molecular, and cellular levels is opening up enormous opportunities to interrupt and reverse the initiation and progression of the disease and provide scientists with numerous targets to arrest by physiological and pharmacologic mechanisms, with the goal of preventing end-stage, invasive disease and impeding or delaying the development of cancer [36]. One of the promising strategies for combating carcinogenesis is to search for substances that can prevent the transformation of normal cells into cancer cells induced by various stimulating factors, e.g., epidermal growth factor (EGF), triphorbol ether (TPA), ultraviolet radiation (UV), etc. The promotion-sensitive mouse epidermal JB6 Cl41 cells are known to respond irreversibly to tumor promoters such as epidermal growth factor (EGF) with induction of anchorage-independent growth in soft agar [37]. Therefore, this well-established culture system was used to study the cancer-preventive activity of rHct-S3. Indeed, the actinoporin delayed the EGF-induced neoplastic transformation of JB6 Cl41 cells (Figure 3a,b) and suppressed colony formation of all cancer cell lines (Figure 3c–e), with the inhibition level of HT-29 and SK-MEL-28 cells comparable to cisplatin. Similar activity was previously demonstrated for RTX-A [16]. This polypeptide prevented malignant transformation of JB6 P^+^ Cl41 cells and suppressed the growth of HeLa cell colonies at nanomolar concentrations [16].

The most significant cancer-preventive activity of rHct-S3 was found in colon cancer cells. Therefore, we examined the effects of rHct-S3 on the migration of colon cancer cells, as well as their proliferation, in order to incorporate the influence of cell proliferation in the interpretation of the results of migration assays. In fact, tumor cell migration essentially contributes to invasion and metastatic spread, ultimately resulting in progression of disease. More than 30% of patients with colorectal carcinoma have clinically detectable metastases at the time of primary diagnosis [38]. Since the most serious complication and the main cause of death of patients with colorectal carcinoma are distant metastases, the evolution of antimetastatic activity of potential therapeutic agents continues to be an important and urgent task. The mechanism of metastases formation is complex and not fully understood. The migration, intravasation, extravasation of cancer cells and formation of a new vessels (neoangiogenesis) to consolidate a secondary tumor at a distant site are the most important steps of the metastasis process [39]. Remarkably, rHct-S3 almost completely suppressed the migration of HT-29 cells at a concentration of 4 μM (Figure 4a,b). Moreover, the actinoporin possessed a moderate antiproliferative activity (Supplementary Figure S1), but its impact on the anti-migratory activity of rHct-S3 was not significant.

During metastasis, the degradation of extracellular matrix (ECM) and components of the basement membrane by proteases facilitates the detachment of cancer cells, their crossing of tissue boundaries, and invasion into adjacent tissue compartments [40]. In recent years, the importance of cancer-associated proteases such as matrix metalloproteinases MMP-2 and MMP-9 in invasion and metastasis has been reported for a variety of solid malignant tumors [41]. Indeed, the actinoporin was found to effectively inhibit an expression of MMP-2 and MMP-9 (Figure 4c) that apparently resulted in the decrease in HT-29 cell migration. Recently, it was shown that caspase-3 is also able to influence the migration and invasion of colorectal cells [42]. In addition, caspase-3 is a key executioner of programmed cell death. In fact, rHct-S3 treatment cleavage of total caspase-3, followed by PARP cleavage, mediate both anti-migratory activity and induction of apoptosis in HT-29 cells (Figure 4d). In line with the pro-apoptotic activity of rHct-S3, an up-regulation of pro-apoptotic Bax and suppression of anti-apoptotic Bcl-2 were observed.

In conclusion, *H. crispa* actinoporin shows promising anticancer activity with a strong inhibiting effect on the migratory potency of cancer cells. We revealed for the first time that the actinoporin was able to inhibit cancer colony formation and cell migration via suppression of MMP-2 and MMP-9 expression and induce cell apoptosis via activation of caspase-3, cleavage of PARP, activation of Bax and suppression of Blc-2 expressions. The results indicate a high potential of the actinoporin to prevent cancer disease progression. Deep investigations of the underlying mechanism of the effect on apoptotic PI3K/AKT/mTOR, and of cell adhesion signaling pathways, are still to be performed.

## 4. Materials and Methods

### 4.1. Expression and Isolation of Recombinant Hct-S3

The recombinant plasmid obtained earlier was transformed into *E. coli* strain Rosetta (DE3) (Novagen, Merck KGaA, Darmstadt, Germany). Transformed cells were cultured at 37 °C in 1 L of Luria-Bertani medium containing 50 μg/mL kanamycin (Gibco, Thermo Fisher Scientific, Gaithersburg, MD, USA) until the optical density (OD_600_) ~0.5 was reached. After induction with IPTG at a concentration of 0.2 mM, the cells were incubated at 19 °C for 18 h at 180 rpm, centrifuged for 6 min at 6000 rpm at 4 °C, and supernatant was removed. The presence of rHct-S3 was determined in 12% polyacrylamide gel by Laemmli’s SDS-PAGE method [43]. Precipitate was resuspended in the start buffer for affinity chromatography (400 mM NaCl, 20 mM Tris-HCl buffer, pH 8.0) and disrupted by French-press homogenizer (Thermo Fisher Scientific, Waltham, MA, USA) using the mini-cell (3.7 mL). Lysed cells were centrifuged for 10 min at 10,000 rpm to remove all insoluble particles. Supernatant was applied to a Ni-NTA agarose (Qiagen, Venlo, Netherlands), the fusion protein was purified with 5 volume of wash buffer (400 mM NaCl, 50 mM imidazole, 20 mM Tris-HCl buffer, pH 8.0) and 5 volume of start buffer. The fusion protein was cleaved by enteropeptidase (1 unit/mg protein) at room temperature for 18 h at 80 rpm. The recombinant actinoporin were purified from enteropeptidase on soybean trypsin inhibitor-agarose (Sigma-Aldrich, St. Louis, MO, USA) and desalted on a centrifugal filter tube (Millipore, Lenexa, KS, USA) with capacity < 3000 Da. The molecular masses of the purified rHct-S3 were analyzed by Ultra Flex III MALDI-TOF/TOF mass spectrometer (Bruker, Bremen, Germany). The amino acid sequence of rHct-S3 were determined on an automated sequencer protein Procise 492 Clc (Applied Biosystems, Foster City, CA, USA).

The purified rHct-S3 was dissolved in PBS, filtered by 0.22 µm “Millipore” membranes (Billerica, MA, USA) and used for the bioactivity experiments.

### 4.2. Cell Lines

Normal mouse epidermal cells JB6 Cl41 (ATCC^®^ no. CRL-2010™), human colorectal carcinoma HT-29 cells (ATCC^®^ no. HTB-38™), breast cancer MDA-MB-231 cells (ATCC^®^ HTB-26™), and melanoma SK-MEL-28 cells (ATCC^®^ no. HTB-72™) were obtained from the American Type Culture Collection (Manassas, VA, USA).

### 4.3. Cell Culture

JB6 Cl41, HT-29, MDA-MB-231, and SK-MEL-28 cells were cultured in complete MEM/5% FBS, McCoy’s 5A/10% FBS, DMEM/10% FBS, and MEM/10% FBS medium, respectively, containing 1% penicillin-streptomycin solution. The cell cultures were maintained at 37 °C in humidified atmosphere containing 5% CO_2_. Every 3–4 days, cells were rinsed with PBS, detached from the tissue culture flask by 0.25% trypsin/0.5 mM EDTA, and 10–20% of the harvested cells were transferred to a new flask containing fresh culture media. The cells after 4–5 passages were used for the experiments. The cells were passaged for a month.

### 4.4. MTS Assay

To determine cytotoxic activity of rHct-S3, cells (1.0 × 10^4^) were seeded in 96-well plates (“Jet Biofil”, Guangzhou, China) and cultured in 200 µL of complete culture medium for 24 h at 37 °C in a 5% CO_2_ incubator. The cell monolayer was washed with PBS and treated either with PBS (control) or various concentrations of rHct-S3 (0.01 µM–10 µM) in fresh appropriate culture medium for 24 h. Subsequently, the cells were incubated with 15 µL MTS reagent (“Promega”, Madison, WI, USA) for 3 h, and the absorbance of each well was measured at 490/630 nm using Power Wave XS microplate reader (“BioTek”, Wynusky, VT, USA).

To determine the antiproliferative activity of rHct-S3, cells (0.7 × 10^4^) were seeded in 96-well plates and cultured in 200 µL of complete culture medium for 24 h at 37 °C in a 5% CO_2_ incubator. The cell monolayer was washed with PBS and treated either with PBS (control) or various concentrations of rHct-S3 (1, 2, and 4 µM) in fresh appropriate culture medium for 24, 48, 72, 96 h. Subsequently, the cells were incubated with 15 µL MTS reagent (“Promega”, Madison, WI, USA) for 3 h, and the absorbance of each well was measured at 490/630 nm using Power Wave XS microplate reader (“BioTek”, Wynusky, VT, USA).

### 4.5. The Soft Agar Colony Formation Assay

JB6 Cl41 cells (2.4 × 10^4^) were exposed to EGF (1 ng/mL) and treated with rHct-S3 (1, 2, and 4 µM) in 1 mL of 0.3% Basal Medium Eagle (BME) agar containing 10% FBS, 2 mM L-glutamine, and 25 µg/mL gentamicin. The cultures were maintained at 37 °C in a 5% CO_2_ incubator for 14 days, and the cell’s colonies were scored using a microscope, Motic AE 20 (XiangAn, Xiamen, China) and the ImageJ software.

To estimate the effect of rHct-S3 on colony formation, human cancer cells (2.4 × 10^4^ /mL) were seeded into 6-well plate and treated with rHct-S3 (1, 2, and 4 µM) or cisplatin (3 µM) in 1 mL of 0.3% Basal Medium Eagle (BME) agar containing 10% FBS, 2 mM L-glutamine, and 25 µg/mL gentamicin. The cultures were maintained in a 37 °C, 5% CO_2_ incubator for 14 days, and the cell colonies were scored as described above.

### 4.6. Scratch-Wound Assay

Cell migration assay was performed as previously described [44]. Briefly, HT-29 cells (3 × 10^5^ cells/mL) were seeded into 6-well plates and 24 h later the culture medium was removed and straight scratch was created using a 200 µL sterile pipette tip. Cells were washed twice with PBS to remove cellular debris, replaced with appropriate complete culture media containing rHct-S3 (1, 2, and 4 µM) and incubated for 96 h. All experiments were repeated at least three times in each group. For the image analysis, cell migration into the wound area was photographed at the stages of 0 and 96 h using a microscope, Motic AE 20, and ImageJ software. The cells migration distance was estimated by measuring the width of the wound and expressed as a percentage of each control for the mean of the wound closure area.

### 4.7. Protein Preparation and Western Blotting

The cells (0.5 × 10^6^ cells/well) were seeded in 6-well plates and incubated overnight. The medium was replaced with fresh medium (1 mL/well) containing the investigated actinoporin at the indicated concentrations, and the cells were incubated for the next 48 h. Cells were harvested by scrapers, pelleted (centrifugation, 5 min, 453× *g*), and washed with PBS (10 mL/samples followed by centrifugation, 3 times). Cells were lysed with 70 µL/sample of lysis buffer (0.88% (*w*/*v*) NaCl, 50 mM Tris-HCl (pH 7.6), 1% NP-40 (*v*/*v*), 0.25% (*w*/*v*) sodium cholate, 1 mM PMSF, 1 mM Na_3_VO_4_, containing cOmplete™ EASYpacks protease inhibitors cocktail and PhosSTOP™ EASYpacks phosphatase inhibitors cocktail (Roche, Mannheim, Germany)) on ice for 20 min. Lysates were frozen for 1 h, centrifuged (10 min, 11,170× *g*), the protein-containing supernatants were taken, and the protein concentrations were evaluated using the Bradford assay. Protein extracts were diluted with loading buffer according to the manufacture’s recommendations, heated for 5 min at 99 °C, and subjected to electrophoresis in 4–12.5% gradient SDS-PAGE (Cat. No. 4568083, Bio-Rad, Hercules, CA, USA) at 120 V. A quantity of 20 µg of total protein was loaded to each slot of the gel. Proteins were then transferred from gel to a 0.2 µm pore PVDF membrane (Millipore, Bedford, MA, USA) using the Bio-Rad transfer system (Cat. No. 10026938, Bio-Rad) and the membranes were blocked with 5% (*w*/*v*) BSA in 0.05% Tween-20/TBS. The membranes were consequently treated with the primary and secondary antibodies, according to the manufacturer’s protocol. Signals were detected using the enhanced chemiluminescence system (Thermo Fisher Scientific, Rockford, IL, USA) according to the manufacturer’s protocol. The following primary and secondary antibody were used: anti-caspase-3 (mAb, anti-rabbit, #9662, dilution 1:1000, Cell Signaling), anti-MMP-2 (mAb, anti-rabbit, #13132, dilution 1:1000, Cell Signaling), anti-MMP-9 (mAb, anti-rabbit, #13667, dilution 1:1000, Cell Signaling), anti-PARP (pAb, anti-rabbit, #9542, dilution 1:1000, Cell Signaling), anti-Bcl-2 (pAb, anti-rabbit, #2876, dilution 1:1000, Cell Signaling), anti-α-tubulin (mAb, anti-mouse, T5168, dilution 1:5000, Sigma-Aldrich), anti-Bax (mAb, anti-rabbit, #5023, dilution 1:1000, Cell Signaling),anti-β-actin (mAb, anti-mouse, #CP01, dilution 1:10,000, Calbiochem), secondary anti-mouse IgG-HRP (sheep, #NXA931, dilution 1:10,000, GE Healthcare), secondary anti-rabbit IgG-HRP (goat, #7074, dilution 1:5000, Cell Signaling).

### 4.8. Statistical Analysis

All assays were performed in at least three independent experiments. Results are expressed as the mean ±standard deviation (SD). Student’s t test was used to evaluate the data with the following significance levels: * *p* < 0.05, ** *p* < 0.01, *** *p* < 0.001.

## Supplementary Materials

The following are available online, Figure S1: The effect of rHct-S3 on proliferation of human colorectal carcinoma HT-29 cell lines.
