# Supplementary material for: Sea Anemone Heteractis crispa Actinoporin Demonstrates In Vitro Anticancer Activities and Prevents HT-29 Colorectal Cancer Cell Migration
Source: Molecules. 2020 Dec 17;25(24):5979. doi: 10.3390/molecules25245979 (PMC7766076; doi:10.3390/molecules25245979)
Supplement: Supplementary file 1 [file molecules-25-05979-s001.pdf]

Sea Anemone *Heteractis crispa* Actinoporin Demonstrates In Vitro Anticancer Activities and Prevents HT-29 Colorectal Cancer Cell Migration

Aleksandra Kvetkina [1,*,†], Olesya Malyarenko [1,†], Aleksandra Pavlenko [1], Sergey Dyshlovoy [2,3,4], Gunhild von Amsberg [2,3], Svetlana Ermakova [1], and Elena Leychenko [1]

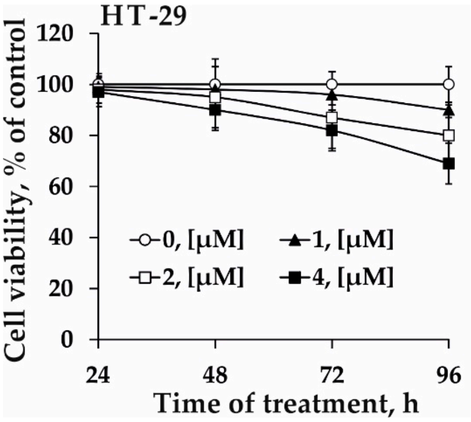

Figure S1. The effect of rHct-S3 on proliferation of human colorectal carcinoma HT-29 cell lines. The inhibiting activity was determined by MTS assay after 24, 48, 72, and 96 h of treatment. The results are expressed as the percentage of inhibition that produced a reduction in absorbance by rHct-S3 treatment at concetnration of 1 μM, 2 μM, and 4 μM compared the non-treated cells (0 μM). Results are expressed as the mean ± standard deviation (SD).
